# Immuno-Stimulating Activity of 1,25-Dihydroxyvitamin D in Blood Cells from Five Healthy People and in Blasts from Five Patients with Leukemias and Pre-Leukemic States

**DOI:** 10.3390/ijms24076504

**Published:** 2023-03-30

**Authors:** Aleksandra Marchwicka, Kuba Nowak, Anastasiia Satyr, Dariusz Wołowiec, Ewa Marcinkowska

**Affiliations:** 1 Department of Biotechnology, University of Wrocław, Joliot-Curie 14a, 50-383 Wrocław, Poland; 2 Faculty of Mathematics and Computer Science, University of Wrocław, Joliot-Curie 15, 50-383 Wrocław, Poland; 3 Institute of Bioorganic Chemistry, Polish Academy of Sciences, Noskowskiego 12/14, 61-704 Poznan, Poland; 4 Department of Hematology, Blood Neoplasms and Bone Marrow Transplantation, Wrocław Medical University, Pasteura 4, 50-367 Wrocław, Poland

**Keywords:** 1,25-dihydroxyvitamin D, vitamin D receptor, exon, transcription, sequencing, blood cells, hematological malignancy, immunity

## Abstract

(1) Hematological malignancies are characterized by an immortalization, uncontrolled proliferation of blood cells and their differentiation block, followed by the loss of function. The primary goal in the treatment of leukemias is the elimination of rapidly proliferating leukemic cells (named blasts). However, chemotherapy, which removes proliferating blasts, also prevents the remaining immune cells from being activated. Acute leukemias affect elderly people, who are often not fit to survive aggressive chemotherapy. Therefore, there is a need of milder treatment, named differentiation therapy, which might simulate the immune system of the patient. 1,25-Dihydroxyvitamin D, or low-calcemic analogs of this compound, were proposed as supporting therapy in acute leukemias. (2) Bone marrow blasts from patients with hematological malignancies, and leukocytes from healthy volunteers were ex vivo exposed to 1,25-dihydroxyvitamin D, and then their genomes and transcriptomes were investigated. (3) Our analysis indicates that 1,25-dihydroxyvitamin D regulates in blood cells predominantly genes involved in immune response, such as *CAMP* (cathelicidin antimicrobial peptide), *CP* (ceruloplasmin), *CXCL9* (C-X-C motif chemokine ligand 9), *CD14* (CD14 molecule) or *VMO1* (vitelline membrane outer layer 1 homolog). This concerns blood cells from healthy people, as well as blasts from patients with hematological malignancies. In addition, in one patient, 1,25-dihydroxyvitamin D significantly downregulated transcription of genes responsible for cell division and immortalization. (4) In conclusion, the data presented in this paper suggest that addition of 1,25-dihydroxyvitamin D to the currently available treatments would stimulate immune system, inhibit proliferation and reduce immortal potential of blasts.

## 1. Introduction

The vitamin D receptor (VDR) belongs to the family of nuclear receptors, which function as ligand-activated transcriptional regulators [1]. 1,25-Dihydroxyvitamin D (1,25D) is a natural ligand for VDR, and the 1,25D-VDR complex regulates the transcription of many mammalian genes [2]. The best-known function of the 1,25D-VDR system is the maintenance of healthy bones by regulating calcium-phosphate homeostasis [3]. It has been appreciated that 1,25D-VDR complex regulates also immune functions of human body [4]. The ability to perform genome-wide analyses has provided deeper insight into this process [5]. The genome-wide studies performed in cell lines have revealed that among hundreds of VDR-regulated genes, many belong to the immune system [6]. Recent research has documented that there are almost 200 genes directly regulated by liganded VDR in human blood cells, and about 500 secondary targets [7]. The cells that appear to be the most important targets of 1,25D in the immune system are macrophages and dendritic cells. These cells not only have high expression of *VDR* [8], but are also able to produce 1,25D from its precursor 25-hydroxyvitamin D (25D) [9,10]. 1,25D is capable of inducing monocytic markers of differentiation in hematopoietic stem cells (HSCs) [11], and it also directly regulates the expression of genes responsible for macrophage functions, such as *CD14* (encoding co-receptor for lipopolysaccharide) [12], *CAMP* (encoding cathelicidin) [13] or *TNF* (encoding tumor necrosis factor α; TNFα) [14].

1,25D is produced by the human body from 7-dehydrocholesterol, and its effective concentration is strictly regulated by feedback mechanisms. At first, vitamin D is produced from 7-dehydrocholesterol in human skin exposed to UV light. Activation of vitamin D is controlled by cytochrome P450 mixed-function oxidases (CYPs) and occurs in two steps: 25-hydroxylation followed by 1α-hydroxylation [15]. The first stage of activation occurs in the liver, where vitamin D undergoes hydroxylation by 25-hydroxylase (CYP2R1/CYP27A1), converting it to 25D. 25D it is then transported to the kidneys, where it undergoes hydroxylation at C-1 by 1α-hydroxylase (encoded by *CY27B1*) and results in the formation of the active metabolite, 1,25D.

Hematological malignancies are characterized by an immortalization and uncontrolled proliferation of blood cells and their differentiation block, followed by the loss of function of the properly differentiated counterparts [16]. Thus, all leukemias, and especially acute types, are accompanied by an increased risk of infections [17]. The primary goal in the treatment of leukemias is an elimination of rapidly proliferating leukemic cells (named blasts). However, chemotherapy removes not only proliferating blasts but also prevents the remaining immune cells from being activated, since immune activation is always accompanied by proliferation [18]. In this way, chemotherapy-induced immunodeficiency adds to the leukemia-induced immunodeficiency. The need to improve functions of the immune system has been widely appreciated.

This issue prompted us to perform a study in which blasts from the bone marrows of patients with hematological malignancies and blood leukocytes from healthy volunteers were ex vivo exposed to 1,25D, and then the transcriptomes from these cells were sequenced. In parallel, whole exon sequencing (WES) was performed using DNA from these cells.

## 2. Results

### 2.1. Results of Whole Exon Sequencing (WES)

DNA was extracted from patient’s bone marrow or healthy donor’s blood samples and WES was performed by Novogene. Then, InDel (insertion/deletion) analysis was made. These InDels, which were shared between the healthy persons and the patients with hematological malignancies were filtered out. The InDels present only in patients were checked for its functional annotation and the InDels that have functional impact on the encoded proteins, were then taken to the analysis. The list of mutations characteristic for leukemias and pre-leukemic states [19,20,21,22] are presented in Table 1, while the list of all InDels detected in the patients’ malignant cells is given in the Supplementary Table S1. Moreover, the information about the fusion genes expressed in patients’ blasts was read out from RNAseq data and presented in Table 1. Fusion genes and mutations characteristic for leukemias and pre-leukemic states were not present in healthy individuals.

### 2.2. Results of RNA Sequencing (RNAseq)

**Figure 1:**
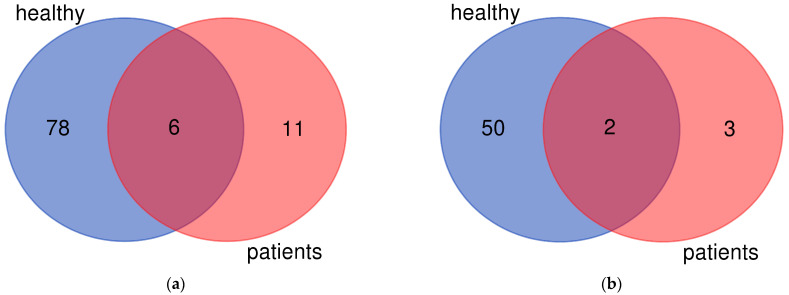
Venn diagrams presenting differentially expressed genes. The overlap between the genes that are upregulated (**a**) or downregulated (**b**) after exposure to 1,25D of the cells from healthy volunteers and from patients. Figure 1 was prepared using an online tool available at https://bioinformatics.psb.ugent.be/webtools/Venn/ (accessed on 26 March 2023).

Leukocytes from healthy people and blast cells from patients were seeded into culture medium and exposed for 96 h to 10 nM 1,25D or the solvent. Total RNA was isolated after the exposure, transcribed to cDNA and RNAseq was performed. Then the genes regulated in response to 1,25D were analyzed. Statistical thresholds of fold change >2 and corrected *p*-value < 0.05 were applied. There were 84 common genes upregulated in all healthy individuals, and 52 common genes downregulated in all healthy individuals. There were also 17 common genes upregulated, and 5 common genes downregulated in all patients (Figure 1). Out of these genes 6 were upregulated in all healthy and all ill people examined (Figure 1a), and 2 downregulated in all healthy and all ill people examined (Figure 1b).

#### 2.2.1. Genes Upregulated after Exposure to 1,25D

**Figure 2:**
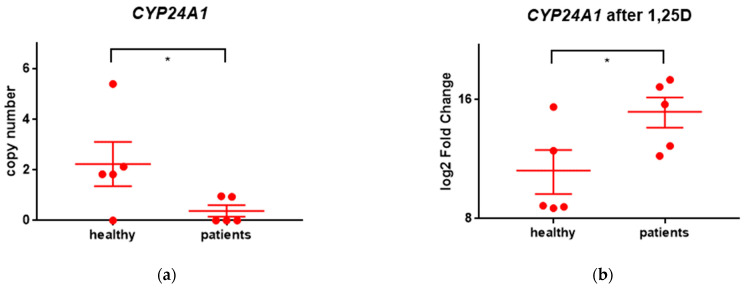
Expression of *CYP24A1* in leukocytes from all individuals. Leukocytes from healthy volunteers and blasts from patients were ex vivo exposed to a vehicle or 10 nM 1,25D for 96 h. Then the mRNA was isolated, transcribed to cDNA and sequenced. The background expression (**a**) and the change in expression of *CYP24A1* between 1,25D-treated sample in comparison to vehicle-treated sample (**b**) were presented as a dot for everyone. The graph presents means ± SEM and the values obtained for everyone. Values that differ significantly between patients and healthy people are marked with whiskers and asterisks.

CYP24A1 is a mitochondrial enzyme catalyzing hydroxylation reactions that lead to the degradation of 1,25D. Subsequent hydroxylations of the side chain produce calcitroic acid which is excreted in the bile [23]. Transcription of the *CYP24A1* gene is upregulated after binding the 1,25D to VDR [24]. In all individuals included in our study, *CYP24A1* was strongly upregulated after exposure to 1,25D. The upregulation was significantly higher in patients than in healthy individuals (Figure 2b). The basal expression of *CYP24A1* in untreated cells was low in all individuals, and significantly lower in patients than in healthy people (Figure 2a).

The other genes upregulated by 1,25D in all individuals were the ones encoding cathelicidin antimicrobial peptide (*CAMP*), vitelline membrane outer layer 1 homolog (*VMO1*), PDZ and LIM domain 4 (*PDLIM4*), ceruloplasmin (*CP*) and parvalbumin (*PVALB*). The data presented in Table 2 indicate that the weakest upregulation was about 2 times in case of *VMO1* and *PDLIM4*, while the strongest upregulation was about 2^18^ (~260,000) times in case of *CYP24A1* and *CP*.

The list of the genes that were upregulated in all healthy volunteers, and the list of the genes that were upregulated in all patients are presented in the Supplementary Table S2a,b.

#### 2.2.2. Genes Downregulated after Exposure to 1,25D

There were two genes downregulated in all individuals examined in our study. These were *CXCL9* which encodes C-X-C motif chemokine ligand 9 and *STEAP1B* encoding STEAP family member 1B (Table 3).

The list of the genes that were downregulated in all healthy volunteers, and the list of the genes that were downregulated in all patients are presented in the Supplementary Table S2c,d.

#### 2.2.3. Cellular Processes Activated after Exposure of the Cells to 1,25D

Eventually, we were interested which biological processes (BPs) were the most enriched in the cells exposed to 1,25D in comparison with control cells. For that purpose, we used a NovoSmart software component, which has been based on the Gene Ontology database (http://www.geneontology.org/ accessed on 26 March 2023). We analyzed 25 most enriched BPs in each individual examined in our study. In nine out of ten people, the most enriched processes were related to immunity (see Supplementary Figure S1a as an example). There was however one patient, in whose cells the BPs most enriched after exposure to 1,25D were connected to cell cycle regulation (Supplementary Figure S1b).

## 3. Discussion

The results from in vitro studies provided good support to the use of 1,25D to treat leukemia [25]. However, the results of small clinical trials with 1,25D against MDS or AML were inconclusive [26]. Now it seems obvious that 1,25D, or its low calcemic analogs, will not become sole treatment against hematological malignancies, but might be used in combination treatments [27]. The immuno-stimulating function of 1,25D in hematological malignancies, which are always accompanied by immune dysfunction, is of primary importance. This was why we wanted to address in this study the regulatory potential of 1,25D in blasts from patients with hematological malignancies, and to compare them to healthy individuals.

There were 84 common genes upregulated in all healthy individuals, and 52 common genes downregulated in all healthy individuals. There were also 17 common genes upregulated, and 5 common genes downregulated in all patients (Figure 1). We will now analyze the biological roles of these genes that were similarly regulated in all individuals:

***CYP24A1*** encodes 24-hydroxylase of 1,25D, which is localized in the inner membrane of mitochondria, and effectively regulates the concentration of this very active compound. The gene encoding CYP24A1 is one of the most strongly regulated VDR-target genes, and its promoter contains multiple vitamin D response elements (VDREs) [24]. Multiple copies of the gene have been detected in colon cancer, thus, *CYP24A1* has been proposed to be a tumor suppressor [28,29]. *CYP24A1* was strongly upregulated in all individuals included in our study. This was most probably caused by low background levels of *CYP24A1* in all individuals (Figure 2a). However, as presented in Figure 2 the upregulation in healthy individuals was significantly lower than in patients. There might be various reasons of such difference. One possible reason is that healthy people have somehow higher background levels of vitamin D in their bloodstream, therefore constitutive expression of *CYP24A1* in these people is higher than in sick ones. The results presented in Figure 2a indicate at significant difference in background levels of *CYP24A1*.

***CAMP*** gene encodes cathelicidin antimicrobial peptide. This gene has been identified as directly regulated by 1,25D/VDR [13] and has been known as one of the most strongly regulated by 1,25D [2]. The function of cathelicidin is in innate immune system. The CAMP gene encodes the 18 kDa precursor of human cationic antimicrobial protein LL-37, which disrupts the membranes of pathogens [30]. In addition to direct anti-bacterial action of LL-37, cathelicidin takes part in the regulation of macrophage, dendritic cells, and T-cells functions [31].

***CP*** encodes ceruloplasmin, which is the main copper-binding glycoprotein, carrying about 40–70% of total copper in blood plasma [32]. Ceruloplasmin in addition to its copper-transporting role is an enzyme which oxidates Fe^2+^ into Fe^3+^. Moreover, ceruloplasmin is one of the main extracellular radical scavengers [32]. Copper is not only an essential micronutrient, but it can be directly toxic towards some microorganisms. The accumulation of copper in macrophages has been shown in some bacterial infections [33]. The upregulation of *CP* gene in patients treated with vitamin D has been reported already [34].

***PDLIM4*** encodes PDZ and LIM domain 4. PDZ-LIM4 belongs to the group of proteins that play diverse biological roles. PDZ-LIM proteins act as adapters recruiting signaling molecules to the actin cytoskeleton, and they are involved in bone development. Certain variants of the *PDLIM4* gene have been associated with osteoporosis [35]. However, the role of PDZ-LIM4 as a tumor suppressor in prostate cancer has been also suggested [36].

***PVALB*** encodes parvalbumin, an acidic calcium-binding protein with low molecular weight. Parvalbumin has been believed to be a slow calcium buffer, however, more recent research revealed its protective roles in muscles and gamma-aminobutyric acid neurons [37].

***VMO1*** encodes vitelline membrane outer layer 1 homolog, which can be found in human tears [38]. This protein interacts with lysozyme C and stabilizes tear fluid [39]. Lysozyme C, on the other hand, is an antimicrobial enzyme that breaks down peptidoglycans in walls of gram-positive bacteria [39].

***CXCL9*** which was downregulated in all individuals involved in this study, encodes C-X-C motif chemokine ligand 9 (CXCL9). CXCL9 belongs to the big family of chemokines, small proteins which interact with G protein-coupled receptors, play key roles in chemotaxis and cause extravasation of leukocytes towards the site of inflammation. CXCL9 mediates chemotaxis of cells through receptor CXCR3, which usually is expressed on T cells [40]. CXCR3 serves as a receptor for three chemokines: CXCL9, CXCL10 and CXCL11. In response to pathogen invasion and at the early steps of tumorigenesis these three chemokines regulate immune cell migration, differentiation, and activation, leading to pathogen elimination or tumor suppression. However, at the later steps of tumorigenesis the same chemokines, acting in an autocrine manner, may lead to tumor growth and metastasis [41]. Downregulation of *CXCL9* by VDR has been reported before [2].

***STEAP1B*** gene encodes member 1B, which belongs to the 6-transmembrane epithelial antigen of prostate (STEAP) family. In contrast to the other members of STEAP family, STEAP1 is not a metalloreductase. However, the localization of STEAP1 in vicinity of transferrin and transferrin receptor 1 suggests that STEAP1 plays some role in iron metabolism. Noteworthy, STEAP1 is overexpressed in several types of human cancers [42]. Another member of this family, namely *STEAP4*, has been already reported as downregulated by VDR [2].

As presented in the Table S2a–d, the blood cells from healthy people responded to 1,25D in a more homogenous way than the blasts from patients. There were 84 genes upregulated, and 52 genes downregulated in all healthy volunteers, while only 17 genes upregulated, and 5 genes downregulated in all patients. This difference is understandable, as the genetic background in cells from healthy people is more homogenous than in patients’ blasts. Noteworthy, the mutations responsible for the origin of leukemias affect immune cells, which are target cells for 1,25D. Among the genes upregulated in all healthy people were such as *CD14* [12], *MRC2* encoding mannose receptor C type 2 [43], or *MARCO* encoding macrophage receptor with collagenous structure [44] which are important for proper macrophage function. Many genes connected with the immune system were also downregulated in all healthy people, such as *IL6* (encoding interleukin 6), which stimulates antibody production [45], or *IDO2*, which encodes indoleamine 2,3-dioxygenase 2, an enzyme that inhibits NK and T cell-induced cytotoxicity [46]. Surprisingly, the genes encoding components of the complement element 1q, *C1QA* (complement C1q A chain), *C1QB* (complement C1q B chain) and *C1QC* (complement C1q C chain) were also downregulated by 1,25D [47].

Most of the genes described above encode proteins connected with the immune functions. The general picture emerging from this regulation is consistent with former findings that 1,25D-VDR stimulates innate, and modulates acquired immune response [48]. However, downregulation of *C1QA*, *C1QB* and *C1QC* evades this general scheme. We suppose that upregulation of innate immunity in patients with hematological malignancies might be beneficial for their health.

Out of all patients involved in this study, the blasts from patient No 5 (P5) reacted in a somehow different way to exposure to 1,25D, than blasts of remaining patients and leukocytes from healthy people. This patient had CMML with mutations in *TET2* and *RUNX1* genes. In the blasts and monocytoid cells present in the bone marrow of P5, in addition to upregulation of innate immunity, 1,25D downregulated many genes responsible for cell cycle control. The list of the most strongly downregulated (more than 8 times) genes in P5 is in Supplementary Table S3. Among the genes downregulated by 1,25D in blasts from P5 there are important regulators of cell division such as: *TERT* encoding telomerase reverse transcriptase, *ARC* encoding activity-regulated cytoskeleton-associated protein, *KIF20A* encoding kinesin family member 20A, *CDC20* encoding cell division cycle 20, *CDC25A* encoding cell division cycle 25A, *SKA3* encoding spindle and kinetochore associated complex subunit 3 or *CCNB2* encoding cyclin B2. In addition, many genes responsible for cell invasion, such as *MMP3*, *MMP1*, *MMP10* or *MMP12* which encode metallopeptidases, or *PRTN3* and *PRSS57* which encode proteases have been also strongly downregulated.

We are aware that the most important limitation of this report is the size of both groups: healthy individuals and patients. Our results indicate that blood cells of healthy people respond in rather homogenous manner to 1,25D, while there are bigger differences among patients’ cells. The study on bigger groups of patients with similar mutations in their blast cells should follow this report.

## 4. Materials and Methods

### 4.1. Isolation of Leukocytes

The study population comprised of 5 patients diagnosed with blood malignancies (one lymphoid and 4 myeloid), and 5 healthy people. The patients presented to the Department of Hematology, Blood Neoplasms and Bone Marrow Transplantation, Wrocław Medical University between March 2021 and February 2022 and gave informed consent for this study. The data of all individuals involved in this study are presented in Table 4.

Please note that one of the patients (P3) admitted to the clinics appeared to be healthy after bone marrow examination and was further included into the group of healthy individuals. The study was accepted by the local Ethical Committee (No 866/2020). Isolation of mononuclear cells from patient’s bone marrow or peripheral blood from healthy volunteers was performed as follows: bone marrow or blood was diluted with phosphate-buffered saline (PBS) in 1:1 ratio, carefully layered onto the equal volume of LSM 1077 (PAA LaboratoriesGmbH, Pasching, Austria), and centrifuged at 400× *g* for 30 min at room temperature. The opaque interface was transferred into the fresh sterile tube and washed three times with PBS. The cells were transferred to RPMI 1640 medium at the density of 10^6^ cells/mL, supplemented with 10% FCS, 100 units/mL penicillin and 100 µg/mL streptomycin, and grown in a humidified atmosphere of 95% air and 5% CO_2_ at 37 °C.

### 4.2. Isolation of DNA

DNA was extracted from the patient’s bone marrow or healthy donor’s blood samples, each of a total of 10 archived specimens. DNA was extracted using Monarch^®^ Genomic DNA Purification Kit (New England Biolabs, Ipswich, MA, USA), following the manufacturer’s instructions. The resulting DNA samples were checked for quantity and quality using NanoDrop ND-1000 spectrophotometer (NanoDrop Technologies, Wilmington, DE, USA).

### 4.3. Isolation of mRNA

For RNAseq analyses, the isolated leukocytes were exposed to 10 nM 1,25D or to the solvent for 96 h. RNA was isolated using Fenozol (A&A Biotechnology, Gdańsk, Poland) with the Total RNA Mini kit (A&A Biotechnology) according to the manufacturer’s instructions. RNA concentration and quality were determined spectrophotometrically with a NanoDrop ND-1000 spectrophotometer.

### 4.4. Sequencing

Whole exome sequencing (WES) was performed by Novogene Technology Co., Ltd. (Cambridge, UK). The Agilent SureSelect Human All Exon V6 Kit was used for library construction and capture experiments. The Illumina NovaSeq platform was used for sequencing according to the effective concentration of the library and the data output requirements. High-throughput paired-end sequencing was performed (paired-end 150 bp, PE150).

In RNAseq technique, the single-stranded mRNA was selectively captured or enriched and converted to complementary cDNA for library preparation. RNA quantity was determined using an Agilent 2100 Bioanalyser and NanoDrop ND-1000 spectrophotometer. cDNA libraries are sequenced using the state-of-the-art Illumina NovaSeq platforms, which utilize a paired-end 150 bp sequencing strategy (short-reads).

### 4.5. WES Analysis

The data preprocessing included the following steps. First, raw reads underwent quality control and filtering. Second, the preprocessed reads were mapped with the hg38 reference genome [49]. Next, the information about SNP and InDel variants was called and functionally annotated. All the preprocessing analysis was performed by Novogene.

The InDel analysis was performed using Python libraries (NumPy and Pandas), custom Python script and manual correction. The information about InDels which occur only in exones, was extracted. These InDels, which were shared between the healthy people and patient’s group, were filtered out. The obtained InDels (present in patient’s group), where checked for its functional annotation and the InDels that have functional impact on the encoded proteins were taken to the analysis.

### 4.6. RNAseq Analysis

The initial analysis was performed by Novogene and consisted of data quality control, mapping to the reference genome [49], gene expression quantification, differential expression analysis and functional analysis. Gene expression level was estimated by the abundance of transcripts (count of sequencing) that mapped to genome or exon. Differential expression analysis was performed for control and 1,25D-treated samples from the same individuals. If a gene differed more than twice as much in expression in both sets of samples, it was considered differentially expressed, in case that *p*-value was less than 0.05. Then Gene Ontology enrichment analysis was performed to assess which BPs were enriched after exposure to 1,25D. All the above analysis was performed using NovoSmart software.

In addition, to estimate differences between groups of healthy individuals and patients, a Python 3.10.9 script was prepared to post-process FPKM (expected number of Fragments Per Kilobase of transcript sequence per Millions base pairs sequenced) normalized expression data delivered by Novogene. To get numerical stability, if the gene expression was equal to 0, ε ≈ 10^−4^ was added (the smallest non-zero FPKM expression in the original data was equal to 0.000593).

For each gene ‘g’ it was checked whether its expression was downregulated, upregulated, or left without changes. It was assumed that expression is downregulated if expression after exposure to 1,25D was minimum two times less than in control sample. Analogically it was assumed that ‘g’ is upregulated if expression after exposure to 1,25D was minimum two times larger than in control sample. Formula used:

change = log2 (f_g,125D/f_g,contr)

Having calculated changes for each gene it was possible to find genes that are consistently up- or downregulated in the group of patients, by checking if for each person in group the same change in expression was observed. To make the above analysis possible, the following packages were used: Numpy [50] 1.23.4, SciPy 1.10.1 [51] and Pandas [52] 1.5.2.

### 4.7. Statistical Analysis

A statistical test for each gene independently was performed to check if expression of this gene was significantly changed after adding 1,25D. The Wilcoxon signed-rank test was used, where a null hypothesis that FPKM-s for the group of people (ill or healthy) come from the same distribution in control and treated sample was verified. Null hypothesis was rejected when *p*-value was <0.05.

## 5. Conclusions

In conclusion, the data presented in this paper indicate that the addition of 1,25D to the currently available treatments for hematological malignancies may stimulate immune response and may be useful for the patients. Furthermore, in certain patients, 1,25D may block the proliferation of blasts and reduce their immortal potential. Study on larger groups of patients should follow our report.

## Figures and Tables

**Table 1:** List of mutations and fusion genes characteristic for leukemias detected in patient’s genomes.

Patient No	Diagnosis	Mutated Gene *	Fusion Gene **
P1	Acute lymphoblastic leukemia (ALL)		*TRDV2-TRAC* *BCR-ABL1* *MTAP-CDKN2B-AS1*
P2	Acute myeloid leukemia (AML)		*NOP53-DHX34*
P4	Myelodysplastic syndrome (MDS)	*TET2*	
P5	Chronic myelomonocytic leukemia (CMML)	*TET2*, *RUNX1*	
P6	Acute myeloid leukemia (AML)		*RUNX1-RUNX1T1*

* Detected in WES; ** detected in RNAseq. *TET2* (tet methylcytosine dioxygenase 2), *RUNX1* (RUNX family transcription factor 1), *TRDV2-TRAC* (T cell receptor delta variable 2—T cell receptor alpha constant), *BCR-ABL1* (BCR activator of RhoGEF and GTPase—ABL proto-oncogene 1), *MTAP-CDKN2B-AS1* (methylthioadenosine phosphorylase—cyclin dependent kinase inhibitor 2B), *NOP53-DHX34* (NOP53 ribosome biogenesis factor—DExH-box helicase 34), *RUNX1-RUNX1T1* (RUNX family transcription factor 1—RUNX1 partner transcriptional co-repressor 1).

**Table 2:** Genes upregulated after exposure to 1,25D in the cells from all individuals.

Gene Name	Gene Description	Minimum Upregulation *	Maximum Upregulation *
*CAMP*	cathelicidin antimicrobial peptide	1.3	9.5
*CP*	ceruloplasmin	1.5	17.5
*CYP24A1*	cytochrome P450 family 24 subfamily A member 1	8.5	18.0
*PDLIM4*	PDZ and LIM domain 4	1.0	11.5
*PVALB*	parvalbumin	1.7	14.2
*VMO1*	vitelline membrane outer layer 1 homolog	1.0	5.9

* log2 of Fold Change is presented.

**Table 3:** Gene downregulated after exposure to 1,25D in the cells from all individuals.

Gene Name	Gene Description	Minimum Downregulation *	Maximum Downregulation *
*CXCL9*	C-X-C motif chemokine ligand 9	−1.0	−13.3
*STEAP1B*	STEAP family member 1B	−1.2	−8.6

* log2 of Fold Change is presented.

**Table 4:** Characteristics of the individuals involved in the study.

Patient	Sex	Age	Diagnosis	Blasts
P1	F	64	Acute lymphoblastic leukemia (ALL)	88.6%
P2	F	18	Acute myeloid leukemia (AML)	77%
P3	F	33	Healthy	-
P4	M	74	Myelodysplastic syndrome (MDS) *	3%
P5	M	86	Chronic myelomonocytic leukemia (CMML)	2.6% **
P6	M	37	AML	23.8%
H1	F	30	Healthy	-
H2	F	38	Healthy	-
H3	M	33	Healthy	-
H4	F	32	Healthy	-

* developed after essential thrombocythemia; ** monocytoid cells 55.2%.

## Data Availability

The raw data and the software which were used for analysis presented in this paper can be found at: https://doi.org/10.5281/zenodo.7725307, https://doi.org/10.5281/zenodo.7716053, https://doi.org/10.5281/zenodo.7736064 and https://doi.org/10.5281/zenodo.7740144.

## Supplementary Materials

The following supporting information can be downloaded at: https://www.mdpi.com/article/10.3390/ijms24076504/s1.

## References

[1] Aranda A., Pascual A. (2001). Nuclear hormone receptors and gene expression. Physiol. Rev..

[2] Hanel A., Veldhuizen C., Carlberg C. (2022). Gene-Regulatory Potential of 25-Hydroxyvitamin D(3) and D(2). Front. Nutr..

[3] Holick M. (1996). Vitamin D and bone health. J. Nutr..

[4] Van Etten E., Mathieu C. (2005). Immunoregulation by 1,25-dihydroxyvitamin D3: Basic concepts. J. Steroid Biochem. Mol. Biol..

[5] Chun R., Liu P., Modlin R., Adams J., Hewison M. (2014). Impact of vitamin D on immune function: Lessons learned from genome-wide analysis. Front. Physiol..

[6] Wang T.-T., Tavera-Mendoza L.E., Laperriere D., Libby E., Burton MacLeod N., Nagai Y., Bourdeau V., Konstorum A., Lallemant B., Zhang R. (2005). Large-Scale in Silico and Microarray-Based Identification of Direct 1,25-Dihydroxyvitamin D3 Target Genes. Mol. Endocrinol..

[7] Hanel A., Carlberg C. (2022). Time-Resolved Gene Expression Analysis Monitors the Regulation of Inflammatory Mediators and Attenuation of Adaptive Immune Response by Vitamin D. Int. J. Mol. Sci..

[8] Ryynänen J., Seuter S., Campbell M., Carlberg C. (2013). Gene regulatory scenarios of primary 1,25-dihydroxyvitamin D3 target genes in a human myeloid leukemia cell line. Cancers.

[9] Dusso A., Brown A., Slatopolsky E. (2005). Vitamin D. Am. J. Physiol. Ren. Physiol..

[10] Dusso A., Finch J., Brown A., Ritter C., Delmez J., Schreiner G., Slatopolsky E. (1991). Extrarenal production of calcitriol in normal and uremic humans. J. Clin. Endocrinol. Metab..

[11] Grande A., Montanari M., Tagliafico E., Manfredini R., Zanocco Marani T., Siena M., Tenedini E., Gallinelli A., Ferrari S. (2002). Physiological levels of 1alpha, 25 dihydroxyvitamin D_3_ induce the monocytic commitment of CD34+ hematopoietic progenitors. J. Leukoc. Biol..

[12] Gombart A.F., Luong Q.T., Koeffler H.P. (2006). Vitamin D compounds: Activity against microbes and cancer. Anticancer. Res..

[13] Gombart A.F., Borregaard N., Koeffler H.P. (2005). Human cathelicidin antimicrobial peptide (CAMP) gene is a direct target of the vitamin D receptor and is strongly up-regulated in myeloid cells by 1,25-dihydroxyvitamin D3. FASEB J..

[14] Cohen M.L., Douvdevani A., Chaimovitz C., Shany S. (2001). Regulation of TNF-α by 1α,25-dihydroxyvitamin D3 in human macrophages from CAPD patients. Kidney Int..

[15] Prosser D., Jones G. (2004). Enzymes involved in the activation and inactivation of vitamin D. Trends Biochem. Sci..

[16] Sandler D., Ross J. (1997). Epidemiology of acute leukemia in children and adults. Semin. Oncol..

[17] Lehrnbecher T., Averbuch D., Castagnola E., Cesaro S., Ammann R., Garcia-Vidal C., Kanerva J., Lanternier F., Mesini A., Mikulska M. (2021). 8th European Conference on Infections in Leukaemia: 2020 guidelines for the use of antibiotics in paediatric patients with cancer or post-haematopoietic cell transplantation, in 8th European Conference on Infections in Leukaemia. Lancet Oncol..

[18] Dodd K.C., Menon M. (2022). Sex bias in lymphocytes: Implications for autoimmune diseases. Front. Immunol..

[19] Howard C.M., Zgheib N.B., Bush S., DeEulis T., Cortese A., Mollo A., Lirette S.T., Denning K., Valluri J., Claudio P.P. (2020). Clinical relevance of cancer stem cell chemotherapeutic assay for recurrent ovarian cancer. Transl. Oncol..

[20] Yokota T., Kanakura Y. (2016). Genetic abnormalities associated with acute lymphoblastic leukemia. Cancer Sci..

[21] Patnaik M.M., Tefferi A. (2020). Chronic Myelomonocytic leukemia: 2020 update on diagnosis, risk stratification and management. Am. J. Hematol..

[22] Ogawa S. (2019). Genetics of MDS. Blood.

[23] Jones G., Prosser D.E., Kaufmann M. (2014). Cytochrome P450-mediated metabolism of vitamin D. J. Lipid Res..

[24] Vaisanen S., Dunlop T., Sinkkonen L., Frank C., Carlberg C. (2005). Spatio-temporal activation of chromatin on the human CYP24 gene promoter in the presence of 1alpha,25-dihydroxyvitamin D3. J. Mol. Biol..

[25] Studzinski G.P., Harrison J.S., Wang X., Sarkar S., Kalia V., Danilenko M. (2016). Vitamin D Control of Hematopoietic Cell Differentiation and Leukemia. J. Cell Biochem..

[26] Harrison J., Bershadskiy A. (2012). Clinical experience using vitamin D and analogs in the treatment of myelodysplasia and acute myeloid leukemia: A review of the literature. Leuk. Res. Treat..

[27] Marcinkowska E. (2022). Vitamin D Derivatives in Acute Myeloid Leukemia: The Matter of Selecting the Right Targets. Nutrients.

[28] Höbaus J., Hummel D., Thiem U., Fetahu I., Aggarwal A., Müllauer L., Heller G., Egger G., Mesteri I., Baumgartner-Parzer S. (2013). Increased copy-number and not DNA hypomethylation causes overexpression of the candidate proto-oncogene CYP24A1 in colorectal cancer. Int. J. Cancer.

[29] Horváth H., Lakatos P., Kósa J., Bácsi K., Borka K., Bises G., Nittke T., Hershberger P., Speer G., Kállay E. (2010). The candidate oncogene CYP24A1: A potential biomarker for colorectal tumorigenesis. J. Histochem. Cytochem..

[30] Kościuczuk E.M., Lisowski P., Jarczak J., Strzałkowska N., Jóźwik A., Horbańczuk J., Krzyżewski J., Zwierzchowski L., Bagnicka E. (2012). Cathelicidins: Family of antimicrobial peptides. A review. Mol. Biol. Rep..

[31] Alford M.A., Baquir B., Santana F.L., Haney E.F., Hancock R.E.W. (2020). Cathelicidin Host Defense Peptides and Inflammatory Signaling: Striking a Balance. Front. Microbiol..

[32] Linder M.C. (2016). Ceruloplasmin and other copper binding components of blood plasma and their functions: An update. Metallomics.

[33] Besold A.N., Culbertson E.M., Culotta V.C. (2016). The Yin and Yang of copper during infection. JBIC J. Biol. Inorg. Chem..

[34] Claro da Silva T., Hiller C., Gai Z., Kullak-Ublick G.A. (2016). Vitamin D_3_ transactivates the zinc and manganese transporter SLC30A10 via the Vitamin D receptor. J. Steroid Biochem. Mol. Biol..

[35] Chen J., Hong Z., Zhao C., Bi Q., Qiu B. (2019). Associations between polymorphisms of the PDLIM4 gene and susceptibility to osteoporotic fracture in an elderly population of Han Chinese. Biosci. Rep..

[36] Vanaja D.K., Grossmann M.E., Cheville J.C., Gazi M.H., Gong A., Zhang J.S., Ajtai K., Burghardt T.P., Young C.Y.F. (2009). PDLIM4, An Actin Binding Protein, Suppresses Prostate Cancer Cell Growth. Cancer Investig..

[37] Permyakov E.A., Uversky V.N. (2022). What Is Parvalbumin for?. Biomolecules.

[38] Wang Z., Chen Z., Yang Q., Jiang Y., Lin L., Liu X., Wu K. (2014). Vitelline Membrane Outer Layer 1 Homolog Interacts With Lysozyme C and Promotes the Stabilization of Tear Film. Investig. Ophthalmol. Vis. Sci..

[39] Ragland S., Criss A. (2017). From bacterial killing to immune modulation: Recent insights into the functions of lysozyme. PLoS Pathog..

[40] Dyer D.P. (2020). Understanding the mechanisms that facilitate specificity, not redundancy, of chemokine-mediated leukocyte recruitment. Immunology.

[41] Tokunaga R., Zhang W., Naseem M., Puccini A., Berger M.D., Soni S., McSkane M., Baba H., Lenz H.J. (2018). CXCL9, CXCL10, CXCL11/CXCR3 axis for immune activation-A target for novel cancer therapy. Cancer Treat Rev..

[42] Gomes I.M., Maia C.J., Santos C.R. (2012). STEAP proteins: From structure to applications in cancer therapy. Mol. Cancer Res..

[43] Fischer S., Stegmann F., Gnanapragassam V.S., Lepenies B. (2022). From structure to function-Ligand recognition by myeloid C-type lectin receptors. Comput. Struct. Biotechnol. J..

[44] Xing Q., Feng Y., Sun H., Yang S., Sun T., Guo X., Ji F., Wu B., Zhou D. (2021). Scavenger receptor MARCO contributes to macrophage phagocytosis and clearance of tumor cells. Exp. Cell Res..

[45] Tanaka T., Akira S., Yoshida K., Umemoto M., Yoneda Y., Shirafuji N., Fujiwara H., Suematsu S., Yoshida N., Kishimoto T. (1995). Targeted disruption of the NF-IL6 gene discloses its essential role in bacteria killing and tumor cytotoxicity by macrophages. Cell.

[46] Fujiwara Y., Kato S., Nesline M.K., Conroy J.M., DePietro P., Pabla S., Kurzrock R. (2022). Indoleamine 2,3-dioxygenase (IDO) inhibitors and cancer immunotherapy. Cancer Treat. Rev..

[47] Thomas S., Smatti M.K., Ouhtit A., Cyprian F.S., Almaslamani M.A., Thani A.A., Yassine H.M. (2022). Antibody-Dependent Enhancement (ADE) and the role of complement system in disease pathogenesis. Mol. Immunol..

[48] Bikle D.D. (2022). Vitamin D Regulation of Immune Function. Curr. Osteoporos. Rep..

[49] Mortazavi A., Williams B.A., McCue K., Schaeffer L., Wold B. (2008). Mapping and quantifying mammalian transcriptomes by RNA-Seq. Nat. Methods.

[50] Harris C.R., Millman K.J., van der Walt S.J., Gommers R., Virtanen P., Cournapeau D., Wieser E., Taylor J., Berg S., Smith N.J. (2020). Array programming with NumPy. Nature.

[51] Virtanen P., Gommers R., Oliphant T.E., Haberland M., Reddy T., Cournapeau D., Burovski E., Peterson P., Weckesser W., Bright J. (2020). SciPy 1.0: Fundamental algorithms for scientific computing in Python. Nat. Methods.

[52] The Pandas Development Team (2022). Pandas-Dev/Pandas: Pandas (v1.5.2). Zenodo. https://zenodo.org/record/7344967#.ZCUH13ZByUk.

