# Supplementary material for: Immuno-Stimulating Activity of 1,25-Dihydroxyvitamin D in Blood Cells from Five Healthy People and in Blasts from Five Patients with Leukemias and Pre-Leukemic States
Source: Int J Mol Sci. 2023 Mar 30;24(7):6504. doi: 10.3390/ijms24076504 (PMC10094698; doi:10.3390/ijms24076504)
Supplement: Supplementary file 1 [file ijms-24-06504-s001.zip › ijms-2236422-supplementary.pdf]

Supplementary material

Table S1. The list of mutations found in blast cells from all patients.

Chr	Gene name	Protein name
		**P1 ALL**
1	*KISS1*	KiSS-1 metastasis suppressor
2	*GEN1*	Holliday junction 5' flap endonuclease
3	*MUC4*	mucin 4, cell surface associated
5	*GPRIN1*	G protein regulated inducer of neurite outgrowth 1
5	*MROH2B*	maestro heat like repeat family member 2B
5	*RASGRF2*	Ras protein specific guanine nucleotide releasing factor 2
5	*TXNDC15*	thioredoxin domain containing 15
6	*HIST1H4K*	H4 clustered histone 12
7	*IQCA1L*	IQ motif containing with AAA domain 1 like
8	*C8orf34*	chromosome 8 open reading frame 34
10	*JAKMIP3*	Janus kinase and microtubule interacting protein 3
11	*KRTAP5-5*	keratin associated protein 5-5
11	*MUC6*	mucin 6, oligomeric mucus/gel-forming
11	*MUS81*	MUS81 structure-specific endonuclease subunit
12	*C12orf60*	chromosome 12 open reading frame 60
12	*VPS33A*	VPS33A core subunit of CORVET and HOPS complexes
15	*SAXO2*	stabilizer of axonemal microtubules 2
17	*KRTAP1-5*	keratin associated protein 1-5
17	*KRTAP2-2*	keratin associated protein 2-2
19	*ANKLE1*	ankyrin repeat and LEM domain containing 1
19	*DCAF15*	DDB1 and CUL4 associated factor 15
19	*LILRB4*	leukocyte immunoglobulin like receptor B4
19	*SLC27A5*	solute carrier family 27 member 5
19	*ZNF626*	zinc finger protein 626
20	*ARHGAP40*	Rho GTPase activating protein 40
21	*KRTAP10-6*	keratin associated protein 10-6
		**P2 AML**
1	*CD48*	CD48 molecule
1	*CNKSR1*	connector enhancer of kinase suppressor of Ras 1
1	*DENND1B*	DENN domain containing 1B
2	*FTCDNL1*	formiminotransferase cyclodeaminase N-terminal like
3	*MUC20*	mucin 20, cell surface associated
3	*MUC4*	mucin 4, cell surface associated
3	*PPP1R2*	protein phosphatase 1 regulatory inhibitor subunit 2
3	*ZNF717*	zinc finger protein 717
4	*CRIPAK*	cysteine rich PAK1 inhibitor
4	*DCHS2*	dachsous cadherin-related 2
5	*GPRIN1*	G protein regulated inducer of neurite outgrowth 1
5	*PCDHGA3*	protocadherin gamma subfamily A, 3
7	*VWDE*	von Willebrand factor D and EGF domains
8	*FAM86B1*	family with sequence similarity 86 member B1

8	*RP1L1*	RP1 like 1
9	*ANKRD20A1*	ankyrin repeat domain 20 family member A1
9	*IFNK*	interferon kappa
10	*SGPL1*	sphingosine-1-phosphate lyase 1
11	*MUC5AC*	mucin 5AC, oligomeric mucus/gel-forming
11	*UBTFL1*	upstream binding transcription factor like 1
12	*OAS2*	2'-5'-oligoadenylate synthetase 2
13	*SKA3*	spindle and kinetochore associated complex subunit 3
14	*ARHGAP5*	Rho GTPase activating protein 5
15	*C15orf40*	chromosome 15 open reading frame 40
15	*GOLGA6L6*	golgin A6 family like 6
17	*CCDC144A*	coiled-coil domain containing 144A
19	*CARD8*	caspase recruitment domain family member 8
19	*DCAF15*	DDB1 and CUL4 associated factor 15
19	*ZFP30*	ZFP30 zinc finger protein
19	*ZNF417*	zinc finger protein 417
20	*ADAM33*	ADAM metallopeptidase domain 33
20	*EDN3*	endothelin 3
20	*MCM8*	minichromosome maintenance 8 homologous recombination repair factor
22	*CYP2D6*	cytochrome P450 family 2 subfamily D member 6
22	*RIMBP3*	RIMS binding protein 3
X	*VCX3B*	variable charge X-linked 3B
**P4 MDS**		
1	*CFAP74*	cilia and flagella associated protein 74
1	*CYP4B1*	cytochrome P450 family 4 subfamily B member 1
1	*FCN3*	ficolin 3
1	*MRPL37*	mitochondrial ribosomal protein L37
2	*AGAP1*	ArfGAP with GTPase domain, ankyrin repeat and PH domain 1
2	*CFAP221*	cilia and flagella associated protein 221
2	*TNFAIP6*	TNF alpha induced protein 6
2	*WDSUB1*	WD repeat, sterile alpha motif and U-box domain containing 1
3	*MUC20*	mucin 20, cell surface associated
3	*ZNF717*	zinc finger protein 717
4	*AASDH*	aminoadipate-semialdehyde dehydrogenase
4	*TET2*	tet methylcytosine dioxygenase 2
6	*FUCA2*	alpha-L-fucosidase 2
6	*PBOV1*	prostate and breast cancer overexpressed 1
7	*IQCA1L*	IQ motif containing with AAA domain 1 like
7	*MUC3A*	mucin 3A, cell surface associated
7	*NFE2L3*	NFE2 like bZIP transcription factor 3
7	*VWDE*	von Willebrand factor D and EGF domains
8	*FAM86B1*	family with sequence similarity 86 member B1
8	*RP1L1*	RP1 like 1
10	*FRMPD2*	FERM and PDZ domain containing 2
10	*HERC4*	HECT and RLD domain containing E3 ubiquitin protein ligase 4
12	*AKAP3*	A-kinase anchoring protein 3

13	*EPSTI1*	epithelial stromal interaction 1
14	*GMPR2*	guanosine monophosphate reductase 2
16	*PDPK1*	3-phosphoinositide dependent protein kinase 1
16	*C16orf52*	C16orf52 homolog
16	*NPIPB6*	nuclear pore complex interacting protein family member B6
16	*SETD1A*	SET domain containing 1A, histone lysine methyltransferase
17	*P2RX5*	purinergic receptor P2X 5
19	*ZNF418*	zinc finger protein 418
20	*FAM209A*	family with sequence similarity 209 member A
22	*APOL6*	apolipoprotein L6
X	*SLC25A5*	solute carrier family 25 member 5
	**P5 CMML**	
1	*C1orf116*	chromosome 1 open reading frame 116
1	*CYP4B1*	cytochrome P450 family 4 subfamily B member 1
2	*AGAP1*	ArfGAP with GTPase domain, ankyrin repeat and PH domain 1
2	*FASTKD1*	FAST kinase domains 1
2	*MARS2*	methionyl-tRNA synthetase 2, mitochondrial
2	*PASK*	PAS domain containing serine/threonine kinase
3	*HTR3E*	5-hydroxytryptamine receptor 3E
3	*MUC4*	mucin 4, cell surface associated
3	*RETNLB*	resistin like beta
4	*KIAA1109*	KIAA1109
4	*TET2*	tet methylcytosine dioxygenase 2
6	*CAPN11*	calpain 11
7	*AGMO*	alkylglycerol monooxygenase
7	*IQCA1L*	IQ motif containing with AAA domain 1 like
7	*SSPO*	SCO-spondin
9	*TOR2A*	torsin family 2 member A
10	*KLLN*	killin, p53 regulated DNA replication inhibitor
11	*CNTN5*	contactin 5
11	*KCNJ5*	potassium inwardly rectifying channel subfamily J member 5
11	*SLC22A10*	solute carrier family 22 member 10
12	*C3AR1*	complement C3a receptor 1
16	*FHOD1*	formin homology 2 domain containing 1
17	*AMZ2*	archaelysin family metallopeptidase 2
17	*RNF213*	ring finger protein 213
19	*DCAF15*	DDB1 and CUL4 associated factor 15
19	*DMKN*	dermokine
19	*IRGC*	immunity related GTPase cinema
19	*LILRB5*	leukocyte immunoglobulin like receptor B5
19	*TSPAN16*	tetraspanin 16
19	*ZNF626*	zinc finger protein 626
21	*RUNX1*	RUNX family transcription factor 1
X	*RBMX*	RNA binding motif protein X-linked
	**P6 AML**	
1	*CSF3R*	colony stimulating factor 3 receptor

1	*CFHR5*	complement factor H related 5
1	*MAPKAPK2*	MAPK activated protein kinase 2
2	*CCDC121*	coiled-coil domain containing 121
2	*FTCDNL1*	formiminotransferase cyclodeaminase N-terminal like
2	*MOGAT1*	monoacylglycerol O-acyltransferase 1
3	*HTR3E*	5-hydroxytryptamine receptor 3E
3	*ZNF717*	zinc finger protein 717
4	*CRIPAK*	cysteine-rich PAK1 inhibitor
5	*PCDHGA10*	protocadherin gamma subfamily A, 10
7	*MEOX2*	mesenchyme homeobox 2
7	*MUC3A*	mucin 3A, cell surface associated
8	*TEX15*	testis expressed 15, meiosis and synapsis associated
9	*ANKRD20A3*	ankyrin repeat domain 20 family member A1
10	*JAKMIP3*	Janus kinase and microtubule interacting protein 3
10	*SFRP5*	secreted frizzled related protein 5
11	*OR51B5*	olfactory receptor family 51 subfamily B member 5
11	*TRIM64B*	tripartite motif containing 64B
11	*ZNF195*	zinc finger protein 195
12	*AKAP3*	A-kinase anchoring protein 3
12	*GLIPR1L1*	GLIPR1 like 1
12	*PTPRQ*	protein tyrosine phosphatase receptor type Q
13	*SKA3*	spindle and kinetochore associated complex subunit 3
14	*OXA1L*	mitochondrial inner membrane protein
16	*NPIPB6*	nuclear pore complex interacting protein family member B6
16	*SPATA33*	spermatogenesis associated 33
17	*MYO15B*	myosin XVB
17	*TSPAN10*	tetraspanin 10
19	*ETFB*	electron transfer flavoprotein subunit beta
19	*KIR2DS2*	killer cell immunoglobulin like receptor
19	*SIGLEC12*	sialic acid binding Ig like lectin 12
19	*ZNF429*	zinc finger protein 429
19	*ZNF626*	zinc finger protein 626
20	*ABHD16B*	abhydrolase domain containing 16B
22	*APOBEC3H*	apolipoprotein B mRNA editing enzyme catalytic subunit 3H

Table S2a. The genes upregulated in response to 1,25D in blood cells from all healthy volunteers.

Gene name	Protein name	Max upregulation *	Min upregulation *	p **
CD14	CD14 molecule	2.4	1.0	0.03125
CAMP	cathelicidin antimicrobial peptide	9.5	2.1	0.03125
FBP1	fructose-bisphosphatase 1	6.2	1.2	0.03125
ORM1	orosomucoid 1	15.4	3.7	0.03125
G0S2	G0/G1 switch 2	5.8	4.1	0.03125
VSIG4	V-set and immunoglobulin domain containing 4	4.3	2.4	0.03125
SEMA6B	semaphorin 6B	2.9	1.0	0.03125

AQP9	aquaporin 9	3.6	2.0	0.03125
CLEC5A	C-type lectin domain containing 5A	2.4	1.0	0.03125
VMO1	vitelline membrane outer layer 1 homolog	5.9	1.7	0.03125
MARCO	macrophage receptor with collagenous structure	5.2	1.8	0.03125
SLC11A1	solute carrier family 11 member 1	4.1	1.8	0.03125
LRP1	LDL receptor related protein 1	2.8	1.2	0.03125
CCL7	C-C motif chemokine ligand 7	5.5	1.2	0.03125
CA2	carbonic anhydrase 2	3.6	1.4	0.03125
FOS	Fos proto-oncogene, AP-1 transcription factor subunit	1.8	1.1	0.03125
FN1	fibronectin 1	6.4	3.8	0.03125
HBEGF	heparin binding EGF like growth factor	3.0	1.4	0.03125
CYP24A1	cytochrome P450 family 24 subfamily A member 1	15.3	8.5	0.03125
TREM1	triggering receptor expressed on myeloid cells 1	7.0	3.7	0.03125
THBD	thrombomodulin	5.3	2.6	0.03125
MRC2	mannose receptor C type 2	3.7	1.4	0.03125
BCAT1	branched chain amino acid transaminase 1	2.9	1.2	0.03125
PDPN	podoplanin	14.7	3.8	0.03125
LAMC1	laminin subunit gamma 1	2.6	1.1	0.03125
MT1H	metallothionein 1H	6.0	1.0	0.03125
HTRA1	HtrA serine peptidase 1	6.4	3.0	0.03125
ADGRE3	adhesion G protein-coupled receptor E3	6.1	1.4	0.03125
SLAMF9	SLAM family member 9	6.4	1.5	0.03125
STEAP3	STEAP3 metalloreductase	2.2	1.2	0.03125
PDLIM4	PDZ and LIM domain 4	7.6	3.3	0.03125
PID1	phosphotyrosine interaction domain containing 1	7.8	1.6	0.03125
LAMB3	laminin subunit beta 3	3.8	2.3	0.03125
CP	ceruloplasmin	17.5	3.7	0.03125
PVALB	parvalbumin	14.2	5.9	0.03125
CLLU1OS	chronic lymphocytic leukemia up-regulated 1 opposite strand	17.6	1.1	0.03125
CLMN	calmin	2.4	1.5	0.03125
STS	steroid sulfatase	2.5	1.1	0.03125
ZMYND15	zinc finger MYND-type containing 15	3.1	1.0	0.03125
EDN1	endothelin 1	15.1	3.3	0.03125
GPR176	G protein-coupled receptor 176	10.1	1.8	0.03125
BMP6	bone morphogenetic protein 6	4.1	1.1	0.03125
SEMA3C	semaphorin 3C	2.3	2.0	0.03125
RAI14	retinoic acid induced 14	5.7	1.5	0.03125
CYP19A1	cytochrome P450 family 19 subfamily A member 1	12.5	2.8	0.03125
PGBD5	piggyBac transposable element derived 5	11.5	3.2	0.03125
AGRP	agouti related neuropeptide	4.5	2.3	0.03125
UMODL1	uromodulin like 1	9.3	1.8	0.03125
LIPN	lipase family member N	11.0	1.2	0.03125
COL7A1	collagen type VII alpha 1 chain	5.1	1.4	0.03125

PAQR5	progestin and adipoQ receptor family member 5	10.6	1.1	0.03125
TNC	tenascin C	4.0	1.2	0.03125
SLC16A14	solute carrier family 16 member 14	10.2	1.6	0.03125
SLC46A2	solute carrier family 46 member 2	11.4	1.9	0.03125
KCP	kielin/chordin-like protein	10.3	2.0	0.03125
ASAP2	ArfGAP with SH3 domain, ankyrin repeat and PH domain 2	4.2	1.4	0.03125
DBH	dopamine beta-hydroxylase	3.3	1.2	0.03125
IGLON5	IgLON family member 5	11.8	1.1	0.03125
FAIM2	Fas apoptotic inhibitory molecule 2	13.4	1.0	0.03125
PDE2A	phosphodiesterase 2A	5.1	1.2	0.03125
SEZ6L2	seizure related 6 homolog like 2	4.6	1.0	0.03125
KL	klotho	4.2	1.3	0.03125
AOX1	aldehyde oxidase 1	10.9	2.6	0.03125
CATIP	ciliogenesis associated TTC17 interacting protein	2.5	1.3	0.03125
RFPL2	ret finger protein like 2	13.0	1.2	0.03125
COL4A2	collagen type IV alpha 2 chain	3.3	1.0	0.03125
ST18	ST18, C2H2C-type zinc finger	8.1	2.1	0.03125
ITGB8	integrin subunit beta 8	4.4	1.2	0.03125
CLEC3B	C-type lectin domain family 3 member B	9.7	1.0	0.03125
NMNAT2	nicotinamide nucleotide adenylyltransferase 2	10.3	3.4	0.03125
RGMA	repulsive guidance molecule BMP co-receptor a	11.1	1.4	0.03125
RHBDL2	rhomboid like 2	8.9	2.8	0.03125
LGI2	leucine rich repeat LGI family member 2	10.6	1.0	0.03125
MET	MET proto-oncogene, receptor tyrosine kinase	9.1	3.0	0.03125
ZSCAN1	zinc finger and SCAN domain containing 1	10.6	7.4	0.03125
SRRM3	serine/arginine repetitive matrix 3	12.1	1.6	0.03125
WNK2	WNK lysine deficient protein kinase 2	8.2	2.3	0.03125
DSCAML1	DS cell adhesion molecule like 1	10.8	1.6	0.03125
TMEM105	transmembrane protein 105	10.2	3.5	0.03125
CTRC	chymotrypsin C	9.5	1.3	0.03125
CELF5	CUGBP Elav-like family member 5	7.8	1.0	0.03125
VSNL1	visinin like 1	9.4	2.5	0.03125
NEK10	NIMA related kinase 10	5.5	1.3	0.03125
FBN3	fibrillin 3	8.4	2.8	0.03125

* log2 of Fold Change is presented; ** Wilcoxon test.

Table S2b. The genes upregulated in response to 1,25D in blasts from all patients

Gene name	Protein name	Max upregulation *	Min upregulation *	p **
CAMP	cathelicidin antimicrobial peptide	3.3	1.3	0.03125
VMO1	vitelline membrane outer layer 1 homolog	5.9	1.0	0.03125
CYP24A1	cytochrome P450 family 24 subfamily A member 1	18.0	11.5	0.03125
PDLIM4	PDZ and LIM domain 4	11.5	1.0	0.03125
CP	ceruloplasmin	5.2	1.5	0.03125
PVALB	parvalbumin	12.4	1.7	0.03125

ASGR1	asialoglycoprotein receptor 1	2.4	1.2	0.03125
LGALS9C	galectin 9C	3.7	1.8	0.03125
CHST1	carbohydrate sulfotransferase 1	9.9	1.1	0.03125
H3F3C	H3 histone family member 3C	10.0	1.8	0.03125
MFAP2	microfibril associated protein 2	12.0	1.1	0.03125
TBC1D3K	TBC1 domain family member 3K	7.0	1.2	0.03125
PTPRN	protein tyrosine phosphatase, receptor type N	2.1	1.4	0.03125
CRYGN	crystallin gamma N	8.7	1.2	0.03125
PEX5L	peroxisomal biogenesis factor 5 like	8.1	1.4	0.03125
HIST2H3C	histone cluster 2 H3 family member c	8.9	2.1	0.03125
SLC12A1	solute carrier family 12 member 1	8.0	1.7	0.03125

* log2 of Fold Change is presented; ** Wilcoxon test.

Table S2c. The genes downregulated in response to 1,25D in blood cells from all healthy volunteers.

Gene name	Protein name	Min downregulation*	Max downregulation*	p **
C1QC	complement C1q C chain	-2.3	-5.5	0.03125
C1QA	complement C1q A chain	-2.1	-3.7	0.03125
CALHM6	calcium homeostasis modulator family member 6	-1.3	-5.1	0.03125
C1QB	complement C1q B chain	-2.0	-5.3	0.03125
CXCL9	C-X-C motif chemokine ligand 9	-2.2	-6.7	0.03125
CCL13	C-C motif chemokine ligand 13	-4.6	-11.0	0.03125
F13A1	coagulation factor XIII A chain	-1.8	-7.5	0.03125
FOLR2	folate receptor beta	-3.8	-6.6	0.03125
IDO1	indoleamine 2,3-dioxygenase 1	-1.7	-5.0	0.03125
CXCL11	C-X-C motif chemokine ligand 11	-1.3	-7.4	0.03125
HAPLN3	hyaluronan and proteoglycan link protein 3	-1.1	-2.1	0.03125
GGT5	gamma-glutamyltransferase 5	-3.1	-6.4	0.03125
ENPP2	ectonucleotide pyrophosphatase/phosphodiesterase 2	-1.6	-5.7	0.03125
CEACAM3	carcinoembryonic antigen related cell adhesion molecule 3	-1.7	-3.1	0.03125
P2RY14	purinergic receptor P2Y14	-1.1	-5.2	0.03125
GAS6	growth arrest specific 6	-1.1	-2.9	0.03125
CD163L1	CD163 molecule like 1	-2.6	-11.9	0.03125
GFRA2	GDNF family receptor alpha 2	-2.7	-6.7	0.03125
PITX1	paired like homeodomain 1	-2.2	-12.2	0.03125
OLFML3	olfactomedin like 3	-4.0	-14.5	0.03125
IL6	interleukin 6	-2.1	-4.9	0.03125
RARRES1	retinoic acid receptor responder 1	-1.8	-9.9	0.03125
HAMP	hepcidin antimicrobial peptide	-1.2	-4.1	0.03125
KCNJ10	potassium voltage-gated channel subfamily J member 10	-1.3	-4.4	0.03125
KCNJ2	potassium voltage-gated channel subfamily J member 2	-1.4	-3.1	0.03125
CASP5	caspase 5	-2.0	-15.8	0.03125

SAMD4A	sterile alpha motif domain containing 4A	-1.4	-3.9	0.03125
NRG1	neuregulin 1	-2.4	-13.9	0.03125
OR2I1P	olfactory receptor family 2 subfamily I member 1 pseudogene	-1.5	-9.7	0.03125
NEURL3	neuralized E3 ubiquitin protein ligase 3	-1.1	-3.1	0.03125
VCAM1	vascular cell adhesion molecule 1	-1.4	-13.5	0.03125
IL31RA	interleukin 31 receptor A	-1.3	-9.4	0.03125
KCNMA1	potassium calcium-activated channel subfamily M alpha 1	-1.3	-6.8	0.03125
ZNF366	zinc finger protein 366			0.03125
CTTNBP2	cortactin binding protein 2	-1.3	-10.9	0.03125
BCL2L14	BCL2 like 14	-2.3	-13.8	0.03125
P2RY12	purinergic receptor P2Y12	-2.5	-15.2	0.03125
CCL1	C-C motif chemokine ligand 1	-1.4	-9.0	0.03125
STON2	stonin 2	-1.5	-10.8	0.03125
ABLIM3	actin binding LIM protein family member 3	-1.3	-11.6	0.03125
IDO2	indoleamine 2,3-dioxygenase 2	-1.8	-14.0	0.03125
TNFSF18	TNF superfamily member 18	-2.5	-11.8	0.03125
OSBPL6	oxysterol binding protein like 6	-1.7	-8.9	0.03125
KCTD14	potassium channel tetramerization domain containing 14	-1.2	-12.0	0.03125
TRIM54	tripartite motif containing 54	-3.3	-9.4	0.03125
STEAP1B	STEAP family member 1B	-1.2	-1.9	0.03125
IPO4	importin 4	-1.3	-2.6	0.03125
SLC18A1	solute carrier family 18 member A1	-1.2	-11.7	0.03125
TMEM244	transmembrane protein 244	-9.6	-11.7	0.03125
NUAK1	NUAK family kinase 1	-1.2	-10.3	0.03125
AMOTL2	angiomotin like 2	-3.1	-10.8	0.03125
FAM19A4	family with sequence similarity 19 member A4, C-C motif chemokine like	-7.0	-9.7	0.03125

* log2 of Fold Change is presented; ** Wilcoxon test.

Table S2d. The genes downregulated in response to 1,25D in blasts from all patients.

Gene name	Protein name	Min downregulation*	Max downregulation*	p **
CXCL9	C-X-C motif chemokine ligand 9	-1.0	-13.3	0.03125
OLIG2	oligodendrocyte transcription factor 2	-1.5	-11.7	0.03125
ANKRD53	ankyrin repeat domain 53	-1.6	-11.5	0.03125
STEAP1B	STEAP family member 1B	-1.5	-8.6	0.03125
FCAMR	Fc fragment of IgA and IgM receptor	-1.2	-11.7	0.03125

* log2 of Fold Change is presented; ** Wilcoxon test.

Table S3. The most strongly downregulated genes in response to 1,25D in the blasts from P5.

Gene name	Protein name	log2 Fold Change
MMP3	matrix metallopeptidase 3	-10.8
GPRC5A	G protein-coupled receptor class C group 5 member A	-8.1
HS6ST2	heparan sulfate 6-O-sulfotransferase 2	-7.9

MMP1	matrix metallopeptidase 1	-6.4
TNFSF18	TNF superfamily member 18	-5.7
CCL26	C-C motif chemokine ligand 26	-5.5
MMP10	matrix metallopeptidase 10	-5.5
CLDN10	claudin 10	-5.3
HES4	hes family bHLH transcription factor 4	-5.3
C11orf96	chromosome 11 open reading frame 96	-5.1
MMP12	matrix metallopeptidase 12	-5.1
RXFP1	relaxin family peptide receptor 1	-5.0
KLRG2	killer cell lectin like receptor G2	-4.5
FERMT1	fermitin family member 1	-4.4
ALOX15	arachidonate 15-lipoxygenase	-4.3
SYT7	synaptotagmin 7	-4.3
TGM2	transglutaminase 2	-4.2
TERT	telomerase reverse transcriptase	-4.1
TINAGL1	tubulointerstitial nephritis antigen like 1	-4.1
LARP6	La ribonucleoprotein domain family member 6	-4.1
ARC	activity regulated cytoskeleton associated protein	-4.1
CXCL14	C-X-C motif chemokine ligand 14	-4.0
CCL24	C-C motif chemokine ligand 24	-4.0
CHST4	carbohydrate sulfotransferase 4	-4.0
HPDL	4-hydroxyphenylpyruvate dioxygenase like	-4.0
CPNE6	copine 6	-3.9
KIF20A	kinesin family member 20A	-3.7
CD1B	CD1b molecule	-3.6
CXCL5	C-X-C motif chemokine ligand 5	-3.6
NAV3	neuron navigator 3	-3.5
IGLL1	immunoglobulin lambda like polypeptide 1	-3.5
ILDR2	immunoglobulin like domain containing receptor 2	-3.4
MLXIPL	MLX interacting protein like	-3.4
CTNNAL1	catenin alpha like 1	-3.4
GDF15	growth differentiation factor 15	-3.4
PRTN3	proteinase 3	-3.4
CDC20	cell division cycle 20	-3.4
CCL17	C-C motif chemokine ligand 17	-3.4
EPX	eosinophil peroxidase	-3.4
WT1	Wilms tumor 1	-3.4
DLGAP5	DLG associated protein 5	-3.4
OCSTAMP	osteoclast stimulatory transmembrane protein	-3.4
MYBL2	MYB proto-oncogene like 2	-3.4
CGREF1	cell growth regulator with EF-hand domain 1	-3.4
CDC25A	cell division cycle 25A	-3.4
TRH	thyrotropin releasing hormone	-3.4
DKK2	dickkopf WNT signaling pathway inhibitor 2	-3.4
IFI27	interferon alpha inducible protein 27	-3.3
SKA3	spindle and kinetochore associated complex subunit 3	-3.3

CCNB2	cyclin B2	-3.3
GEM	GTP binding protein overexpressed in skeletal muscle	-3.3
CSF1	colony stimulating factor 1	-3.3
BAG3	BCL2 associated athanogene 3	-3.2
MCM10	minichromosome maintenance 10 replication initiation factor	-3.2
SLC7A11	solute carrier family 7 member 11	-3.2
HSPA1B	heat shock protein family A (Hsp70) member 1B	-3.2
NKD2	naked cuticle homolog 2	-3.2
DIAPH3	diaphanous related formin 3	-3.2
FAM222A	family with sequence similarity 222 member A	-3.2
TEAD4	TEA domain transcription factor 4	-3.2
ID1	inhibitor of DNA binding 1. HLH protein	-3.2
CTSG	cathepsin G	-3.2
CDC25C	cell division cycle 25C	-3.2
PLPPR3	phospholipid phosphatase related 3	-3.1
RIBC2	RIB43A domain with coiled-coils 2	-3.1
KIF18A	kinesin family member 18A	-3.1
BIRC5	baculoviral IAP repeat containing 5	-3.1
CCDC3	coiled-coil domain containing 3	-3.1
TPX2	TPX2. microtubule nucleation factor	-3.0
PRSS57	serine protease 57	-3.0
MAD2L1	mitotic arrest deficient 2 like 1	-3.0
IDO1	indoleamine 2.3-dioxygenase 1	-3.0
DEPDC1	DEP domain containing 1	-3.0
HSD11B1	hydroxysteroid 11-beta dehydrogenase 1	-3.0
NPW	neuropeptide W	-3.0
FAM83D	family with sequence similarity 83 member D	-3.0
UBE2C	ubiquitin conjugating enzyme E2 C	-3.0
SH2D4A	SH2 domain containing 4A	-3.0
AUNIP	aurora kinase A and ninein interacting protein	-3.0

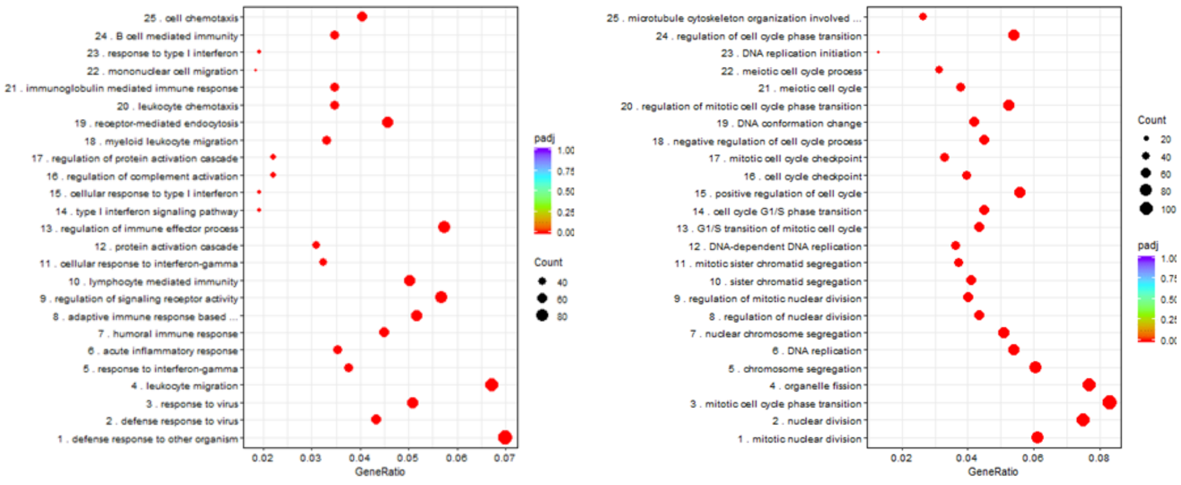

(**a**) (**b**)

Figure S1. Gene expression enrichment after exposure to 1.25D. Peripheral blood leukocytes from a healthy person (**a**) and bone marrow blasts from a patient with chronic myelomonocytic leukemia (CMML) (**b**) were *ex vivo* exposed to 0.1% ethanol (solvent) or 10 nM 1.25D for 96 hours. Afterwards the transcriptomes were sequenced from these samples and analyzed using NovoSmart Software. The graphs present 25 biological processes in which gene expressions were the most enriched in 1.25D-treated samples when compared to the respective controls.
